# Reliability Assessment of Deflection Limit State of a Simply Supported Bridge using vibration data and Dynamic Bayesian Network Inference

**DOI:** 10.3390/s19040837

**Published:** 2019-02-18

**Authors:** Hanbing Liu, Xin He, Yubo Jiao, Xirui Wang

**Affiliations:** 1 College of Transportation, Jilin University, Changchun 130025, China; lhb@jlu.edu.cn (H.L.); xhe16@mails.jlu.edu.cn (X.H.); wangxr17@mails.jlu.edu.cn (X.W.); 2 Key Laboratory of Urban Security and Disaster Engineering of Ministry of Education, Beijing University of Technology, Beijing 100124, China

**Keywords:** structural reliability, bridge deflection, structural health monitoring, modal flexibility, dynamic Bayesian network, Kriging model, artificial bee colony algorithm

## Abstract

Structural health monitoring (SHM) has been widely used in all kinds of bridges. It is significant to accurately assess the serviceability and reliability of bridge subjected to severe conditions by SHM technique. Bridge deflection as an essential evaluation index can reflect structural condition perfectly. In this study, an approach for deflection calculation and reliability assessment of simply supported bridge is presented. Firstly, a bridge deflection calculation method is proposed based on modal flexibility and Kriging method improved by artificial bee colony algorithm. Secondly, a dynamic Bayesian network is employed to evaluate the deflection reliability combined with monitoring results which include modal frequency, mode shape, environmental temperature, and humidity. A linear regression model is established to analyze the relationship between modal parameters and environmental factors. Thirdly, a simply supported bridge is constructed and monitored to verify the effectiveness of the proposed method. The results reveal that the proposed method can precisely calculate the bridge deflection. Finally, the time-dependent reliabilities of two cases are computed and the effects of monitoring factors on bridge deflection reliability are analyzed by sensitivity parameter. It indicates that the reliability is negatively correlated with temperature and more sensitive to mode shape than other three factors.

## 1. Introduction

As one of the important civil infrastructures, bridges are usually subjected to severe conditions caused by environmental effects and repeated vehicles, which affect the serviceability and ultimate capacity of structures seriously [1,2,3]. It will be of great significance to assess the safety and reliability of bridges based on their real-time state information. Owing to the rapid development of sensor technology and computational methods, bridge structural health monitoring (SHM) has been widely used to monitor stress, strain, deflection, modal frequency, mode shape, etc. for all kinds of bridges [4,5,6]. Deflection is one of the key performance evaluation indicators for bridge structure, which can reflect the safety and serviceability condition of bridge perfectly. Therefore, it is essential to assess the serviceability and reliability of bridge utilizing the monitored deflection data.

Nowadays, bridge deflection surveying methods can be mainly classified into two categories: contact type and non-contact type. The first one includes traditional static measurement methods (TSMM), linear variable differential transformers (LVDT) and connected pipe systems (CPS). The other one includes global navigation satellite system (GNSS), photogrammetric techniques (PT), and laser displacement technologies (LDT) [7]. The TSMM is usually carried out through leveling equipment, theodolite and total stations. Although this method can provide accurate measurement results, it is time-consuming and laborious. More importantly, it is unfeasible for long-term deflection monitoring and can only obtain deflections at specific measurement points [8,9]. LVDT is used to measure the differential displacement between the device and measurement target [10]. This system should be free of vibration, which must be installed on a stationary platform. In practice, it is hard to avoid the measurement errors caused by wind, measuring distance, and so on [11]. For CPS, the changes of liquid levels in the connected pipes detected by pressure transmitters are used as the indicators, which can reflect the deflection changes of bridge. Deng et al. [12] utilized this method to monitor a long-span suspension bridge and assessed the serviceability reliability of vertical deflections. The results demonstrated that the CPS presented an excellent capacity to track the deflection under actual traffic conditions. Nevertheless, temperature and vibration will cause the measurement errors. Similar to the LVDT, CPS also needs a stationary platform for reference. The measurement technologies based on GNSS—which includes GPS, GLONASS, Galileo, and BeiDou Navigation Satellite System (under construction)—have been applied to the deflection monitoring of long-span bridges [13]. Moschas et al. [14] expanded the application of GNSS into the monitoring of three-dimensional dynamic deflections of a stiff bridge. However, the drawbacks such as high cost and low accuracy make GNSS impractical for long-term monitoring of bridge structures. PT uses video images to measure the bridge deflection, which is widely used due to its simply installment, cost-effective and relatively precise properties. With the rapid development of vision sensors, the displacement monitoring techniques including digital image correlation, up-sampled cross correlation, phase-based method and some others have been widely applied [11,15]. In fact, they are still limited by the factors such as weather, monitoring distance and low lighting environment in real applications [13]. LDT has the similar operating principle with PT, which is carried out by high fidelity non-contact sensors and provides higher accurate deflection monitoring results. However, the laser beam may have an oblique impact angle on the projection plate because of the laser device and the video camera placed on the same side of the plate. Moreover, strong laser beams equipped for long distance monitoring could have hidden dangers to human health [16].

Meanwhile, the deflection can be calculated by the double integral of the acceleration measured through accelerometers, which are usually used to monitor the modal parameters of bridge [15]. But some researchers reported that this approach was not accurate due to errors in acceleration signals and unknown initial conditions [17]. However, we can make full use of the modal parameters, which are also identified by the accelerometers and widely used as the important monitoring indicators of bridge, to propose a novel bridge deflection calculation method. The modal flexibility matrix calculated by the modal parameters presents the static displacement of arbitrary freedom degree with external force loaded. Therefore, bridge deflection can be obtained by simply multiplying a corresponding force vector with the flexibility. Yi et al. [18] studied an indirect static deflection estimation method utilizing finite element model updating with natural frequencies and mode shapes. It concluded that the deflection can be reasonably estimated by the proposed approach. Tian et al. [19] proposed an ambient vibration test-based deflection prediction method and verified the capability and reliability of this method through field test of a three-span bridge. The results revealed that a good agreement was found between the predicted deflections from the identified flexibility and those measured from the static test. In addition, the accelerometer sensors have advantages of high precision, strong anti-interference, easy to set-up, low cost, and small size, which coincide with the properties of the ideal measurement system. There is no gainsaying that the measurement method based on modal parameters has two shortcomings. Firstly, the deflection measurement precision is influenced by the number of arranged accelerometer sensors. Secondly, only the deflections at sensors positions can be obtained. In this study, the Kriging model [20] is applied to improve the calculation precision based on monitoring results of modal parameters even if a limited number of accelerometer sensors are arranged on bridge. In terms of calculative ability, Kriging model is adopted again to obtain the deflections at arbitrary positions, which is established by the known deflection and position information at this time. Combined with artificial bee colony (ABC) algorithm [21], the Kriging model is updated to improve the deflection calculation precision. 

Hence, the serviceability reliability can be furtherly investigated based on the bridge health monitoring date. In fact, the probabilistic model used for bridge reliability analysis is influenced by the statistical uncertainty. Bayesian network (BN) can realize the improvement of reliability assessment, which is the most useful method for structural reliability assessment by combination of probabilistic model with inspection and monitoring data [22]. Dynamic Bayesian network (DBN) is used to represent evolving time of complex systems, which is an extension of BN with temporal dependency [23]. A few researchers have investigated structural reliability analysis based on Bayesian theory. Boudali et al. [24] proposed a novel reliability modeling and analysis framework based on BN formalism and demonstrated that it was a powerful and suitable reliability analysis framework for dynamic systems. Jacinto et al. [25] took into account the statistical uncertainty on reliability estimates and illustrated that Bayesian methods are especially suitable to deal with this problem. Fan et al. [26] built a Bayesian dynamic nonlinear model with the monitored stress data of the I-39 North Bridge to predict dynamic reliability. Luque et al. [22] developed a computational framework based on DBN to estimate the reliability of deteriorating structural systems with inspection and monitoring results. Chen et al. [27] analyzed the reliability of an existing reinforced concrete structure with monitored information by Bayesian updating. In actual engineering, various reasons can affect both deflection and modal parameters of bridge obviously, for instance, environment factors, concrete creep, and shrinkage [15]. For short-term study, environment temperature and humidity have major impacts on deflection monitoring [28], while concrete creep and shrinkage are negligible. In our previous research [29], we have analyzed the correlations between temperature and modal frequencies. However, how to assess the dynamic reliability of bridge deflection considering temperature and humidity effects is still unknown.

In this study, the bridge deflection monitoring and calculation system based on modal parameter was illustrated, and the deflection calculation process was improved by Kriging method and ABC algorithm. Therefore, bridge deflection can be computed accurately by SHM system of modal parameters, even though bridges are not equipped with static deflection monitoring device. Then, a dynamic reliability analysis approach was presented combining the DBN and the monitoring method proposed in this paper. In addition, a simply supported slab bridge was built to verify the proposed method through the static experiments and a monitoring system was established to collect the corresponding data including modal parameter, environment temperature and humidity for a whole year. Finally, the time-dependent reliability of bridge deflection for two cases was computed and the relationship between the reliability and monitoring factors was analyzed. The sensitivity analysis was carried out to investigate the effects of influence factors on the bridge reliability.

## 2. An Improved Modal-Based Approach to Calculate Bridge Deflection 

### 2.1. Modal Flexibility

Modal flexibility has been widely used for damage identification of bridge, which is calculated by the modal parameters including modal frequency, modal shape (MS) and damping, etc. The modal flexibility matrix of a structure with *n* freedom degree is expressed as [30]

(1) $$F=\varphi \Omega^{-1}\varphi^{T}=\sum_{i=1}^{r}\frac{1}{\omega_{i}^{2}}\varphi_{i}\varphi_{i}^{T},$$

where ***F*** is the modal flexibility matrix; $\varphi =[\varphi_{1};\varphi_{2};\ldots ;\varphi_{r}]$ is the MS matrix, $\varphi_{i}$ represents the *i*th order MS; $\Omega =diag(\omega_{i}^{2})$; $\omega_{i}$ is the *i*th modal frequency; *r* is the number of modal orders.

Arbitrary modal flexibility can be obtained through Equation (1). For instance, the *j*th flexibility is $F_{j}=\sum_{i=1}^{r}\frac{1}{\omega_{i}^{2}}\varphi_{j,i}\varphi_{i}^{T}$, which indicates the static vertical displacements of bridge structure when external force is loaded at node *j*. With the modal order increasing, corresponding modal frequency value will enlarge rapidly. As a result, the lower modal parameters contribute more than the higher ones to the deflection calculation. The higher modal parameters are difficult to be measured accurately. Therefore, we can make a good estimate of the deflections just by a few of the lower modes. In addition, because the contribution coefficient is zero, modal damping does not make contributions to modal flexibility which means modal damping is not related with static bridge deflection. The detailed derivation process can be referred to in Refs. [30,31].

Due to the homogeneity of the characteristic equation, it is impossible to acquire the absolute solution values of MS, which means the MS is unscaled [19]. The MSs obtained by vibration test or theoretical calculation are usually specific node normalized or maximum displacement normalized. These two normalized MSs can be transformed to the mass normalized ones through the modal mass [31], which is expressed as

(2) $$M_{i}=\varphi_{i}M\varphi_{i}^{T},$$

where $M_{i}$ is the *i*th modal mass; $M=diag(m_{j})$ is the mass matrix and $m_{j}$ is the mass of node *j*.

When the modal mass equals to 1, the corresponding MS vector of Equation (2) is the mass normalized. The relationship between the mass normalized mode shapes (MNMS) and others can be written as

(3) $$\varphi_{i}M\varphi_{i}^{T}=\sqrt{M_{i}}{\bar{\varphi }}_{i}M{\bar{\varphi }}_{i}^{T}\sqrt{M_{i}},$$

(4) $${\bar{\varphi }}_{i}=\frac{\varphi_{i}}{\sqrt{M_{i}}},$$

where ${\bar{\varphi }}_{i}$ is the *i*th mass normalized MS vector.

If Equation (4) is substituted into Equation (1), the *j*th modal flexibility will be rewritten as

(5) $${\bar{F}}_{j}=\sum_{i=1}^{r}\frac{1}{\omega_{i}^{2}}{\bar{\varphi }}_{j,i}{\bar{\varphi }}_{i}^{T}.$$

At this point, Equation (5) represents the deflection of bridge structure with unit external force loaded at node *j*. Within the range of elastic deformation, the deflections are proportional to the external force and obey superposition principle. Consequently, we can compute the deflection at the condition of the arbitrary external forces imposed on the bridge structure, which is described as

(6) $$D=\sum_{i=1}^{n}f_{i}{\bar{F}}_{i},$$

where ***D*** is the bridge deflection vector; $f_{i}$ is the force loaded on node *i*.

### 2.2. Improved Modal Flexibility

The shortcoming of calculation method of deflection shown in Section 2.1 is obvious. The deflections at specific nodes can only be computed when the external forces are imposed on the existing nodes. Therefore, it becomes inefficient and uneconomical with the increasing number of sensors arranged on bridge, which is impossible to obtain the deflection at random positions. In this study, an improved modal flexibility-based method is proposed to calculate the deflection of bridge structure through modal parameters. Theoretically, the function form of MS can be expressed as Equation (7). It illustrates that MS is a continuous function, although it does not always satisfy sinusoidal function in actual engineering. Therefore, the needed MSs can be obtained through an interpolation method. To this end, the Kriging method is adopted as the interpolation method in this paper.

(7) $$\varphi_{i}(x)=A\sin\frac{i\pi x}{l},$$

where $\varphi_{i}(x)$ is the *i*th order MS value at arbitrary location *x*; *A* is a constant; *l* is the span length of bridge.

#### 2.2.1. Kriging Model

Kriging interpolation method was firstly proposed for geostatistical applications in 1951 [32]. With development for several decades, it has been widely used in structural reliability analysis [33] or as surrogate model of numerical experiment [34], etc. Kriging model consists of two portions: a realization of a regression model and a statistical process, which is expressed as

(8) $$y(x)=f^{T}(x)\beta +z(x),$$

where y(x) is the deterministic response values for an *m* dimensional input $x=[x_{1},x_{2},\ldots ,x_{m}]$; $f^{T}(x)$ is the shape functions with a vector of *m* polynomial functions $f^{T}(x)=[f_{1}(x),f_{2}(x),\ldots ,f_{m}(x)]$; β is a vector of regression parameters $\beta =[\beta_{1},\beta_{2},\ldots ,\beta_{m}]$, which describes the global trend of the design space; z(x) is a statistical process with mean 0 and variance $\sigma^{2}$, which reflects the local deviations of design space. The covariance between two arbitrary statistical parameters is expressed as

(9) $$\mathrm{cov}[z(x_{i}),z(x_{j})]=\sigma^{2}R(x_{i},x_{j},\theta ),$$

where $R_{i,j}=R(x_{i},x_{j},\theta )$ is the correlation function and an arbitrary element of the correlation matrix, which is described as Equation (9); θ is the tuning parameter, which defines the correlation function.

(10) $$R=\left[\begin{array}{cccc} R_{1,1} & R_{1,2} & \cdots & R_{1,m}\\ R_{2,1} & R_{2,2} & \cdots & R_{2,m}\\ \cdots & \cdots & \ddots & \vdots \\ R_{m,1} & R_{m,2} & \cdots & R_{m,m} \end{array}\right].$$

The mean square error (MSE) of the best linear unbiased estimate is defined as

(11) $$MSE[\hat{y}(x)]=E{\left[C^{T}(x)Y-y(x)\right]}^{2}.$$

Consequently, the Kriging predictor $\hat{y}(x)$ is written as

(12) $$\hat{y}(x)=f^{T}(x)\hat{\beta }+L^{T}(x)R^{-1}(Y-F^{T}\hat{\beta }),$$

where $\hat{\beta }$ is the estimated coefficient matrix of β; $L(x)={\left[R(x,x_{1},\theta ),R(x,x_{2},\theta ),\ldots ,R(x,x_{m},\theta )\right]}^{T}$ is the correlation function between an unknown x and *m* input variables. More details about the Kriging method can be seen in references [35,36].

In this study, the calculation process of deflection based on model flexibility and Kriging method includes two stages. At the first stage, if external force is imposed at the existing node, the deflections can be computed at the nodes directly. Otherwise, the nodes coordinate and the actual measured MSs are chosen as the input and response variables to establish the Kriging model. The needed MSs including the external force position will be obtained by the Kriging model. Then corresponding deflections located at the nodes of the simulated MSs can be calculated through Equations (4)–(6). It should be noticed that the modal mass and mass matrix will be rewritten according to the simulated MSs. If the deflection of target position is not included in the deflections of the first stage, the second stage will start. At the second stage, the deflections calculated at the first stage will be selected as the response variables with the simulated MS nodes coordinate as input variable. Therefore, the deflection at arbitrary location of bridge can be obtained through the Kriging model established at the second stage. 

#### 2.2.2. Artificial Bee Colony Algorithm

Generally, the regression models $f_{i}(x)$ can be fitted by zero order (constant), first order (linear) and second order (quadratic) polynomials [34]. The statistical processes include exponential, Gaussian, linear, and spherical correlation models [37]. The Kriging models built by different tuning parameters, regression and correlation models present different goodness of fitting. The optimal combination can be determined by the artificial swarm intelligence algorithms, such as genetic algorithm (GA), particle swarm optimization (PSO), and artificial bee colony algorithm (ABC) [38]. ABC outperforms other algorithms due to its simple structure, easy implementation and outstanding performance [39,40], which is employed in this paper.

Usually, the original ABC, which was inspired by the cooperative foraging and waggles dance behaviors of honey bee colony, is composed by four phases—namely initialization, employed bees, onlooker bees, and scout bees phases [41]. In this study, the objective function is Euclidean distance between actual mode shape amplitude vector (or the deflection vector) and that one calculated by Kriging model. The tuning parameter *θ* is the solution called as the food source in ABC. The main steps of ABC are summarized as follows:

(1) Initialization phase

At first, an initial food source will be generated by Equation (13) and the fitness can be solved through Equation (14) to evaluate the quality of the food source.

(13) $$\theta_{ij}=lb_{j}+(ub_{j}-lb_{j})\cdot rand(0,1),$$

where $\theta_{ij}$ is the *j*th parameter of the *i*th solution, i=1,2,…,FN, j=1,2,…,D, *FN* and *D* are the number of food source and the dimension number of a solution, respectively; $lb_{j}$ and $ub_{j}$ are the lower and upper bounds of the *j*th parameter, respectively; rand(0,1) is a uniform distributed real number in the interval between 0 and 1.

(14) $$fit_{i}=\{\begin{array}{cc} \frac{1}{1+f(\theta_{i})} & if\;f(\theta_{i})\geq 0\\ 1+abs(f(\theta_{i})) & \mathrm{otherwise} \end{array},$$

where $fit_{i}$ represents the fitness of the *i*th solution; $f(\theta_{i})$ is the objective function value of the food solution $\theta_{i}$.

(2) Employed bees phase

Employed bees will search the vicinity food source, which is defined as Equation (15). The better one will be reserved according to the greedy selection mechanism.

(15) $$v_{ij}=\theta_{ij}+\psi_{ij}\cdot (\theta_{ij}-\theta_{kj}),$$

where $v_{ij}$ is the *j*th parameter of the *i*th new solution; $\theta_{kj}$ is a food source selected randomly in the swarm, and *k* is not equal to *i*; $\psi_{ij}$ is a random number in the range of [−1, 1].

(3) Onlooker bees phase

Employed bees share the nectar information with onlooker bees after they return to the nest. The onlooker bees will choose a food source by its probability shown as Equation (16). The better the food source is, the more the onlooker bees will further exploit it.

(16) $$p_{i}=\frac{fit_{i}}{\sum_{j=1}^{FN}fit_{j}}.$$

(4) Scout bees phase

If the counter of a food source exceeds the control parameter, which means it is exhausted, the employed bee will turn to be a scout bee and search randomly. When the scout bee finds a new solution, it converts back into an employed bee and the counter will be reset to zero. The algorithm will be repeated from Step 2 to Step 4 until the termination criteria is satisfied.

**Figure 1:**
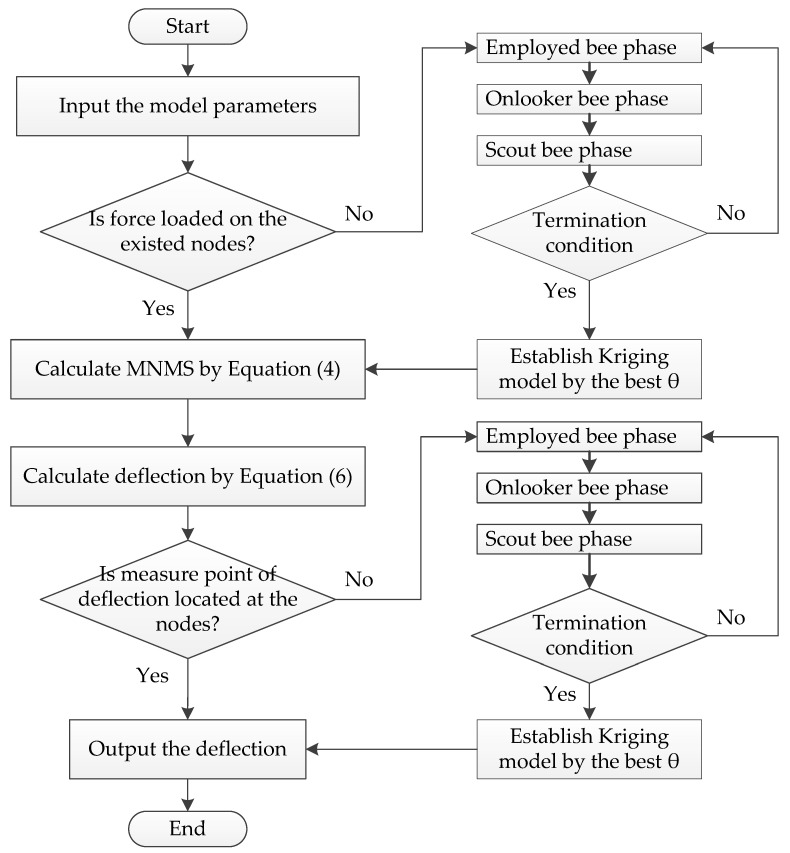
Flowchart of the proposed approach to calculate bridge deflection.

Figure 1 depicts the flowchart of the proposed approach in this paper.

## 3. Dynamic Bayesian Network

### 3.1. Linear Regression Model

Environment factors, such as temperature and humidity, can influence modal frequencies significantly. In Refs. [28,29], they investigated the relations between dynamic properties of bridge and environment factors. They found that the modal frequencies decreased when temperature or humidity increased. Temperature affected modal frequencies more obviously than humidity, whereas both temperature and humidity presented little effect on MSs. In other words, these research results reflect that bridge deflection can be influenced by temperature and humidity based on the modal flexibility theory.

Bayesian methods have been widely used to combine structural reliability evaluation with inspection and monitoring results. Bayesian statistical theory differs from the classical theory since all unknown parameters are considered as random variables. In this paper, the aim of Bayesian theory is to calculate the posterior distribution of modal frequency *Fre* by using the prior probability distribution and the observed results, which is expressed as Ref. [42]

(17) $$f(Fre|X_{Tem},X_{Hum})=\frac{1}{c}L(X_{Tem},X_{Hum}|Fre)\pi (Fre),$$

where $X_{Tem}$ and $X_{Hum}$ represent the corresponding observed data of environment temperature and humidity, respectively; π(Fre) is the prior distribution of *Fre*; $L(X_{Tem},X_{Hum}|Fre)$ is the likelihood function; c is the Bayesian factor calculated as

(18) $$c=\int_{\Omega }L(X_{Tem},X_{Hum}|Fre)\pi (Fre)dFre,$$

where Ω is the parameter space of *Fre*.

For a practical engineering problem, there will be a set of variables needed to be analyzed. These random variables can be described by a statistical model, which consists of three important parts, namely, the response variable *Fre*, the explanatory variables $\left[X_{Tem},X_{Hum}\right]$ and a linking mechanism between the two sets of variables. The model can be written as

(19) $$Fre|X_{Tem},X_{Hum}∼D(\eta ),$$

where D(η) is a distribution family with parameter vector η of *Fre* conditional on the observed variables.

Normal regression models are the common methods to solve this kind of problems [43]. Here, the explanatory variable can be considered as non-stochastic component. Assuming the response variable *Fre* satisfies the normal distribution with mean $\theta_{Fre}$ and variance $\nu_{Fre}$. At this time, Equation (19) can be rewritten as

(20) $$Fre|X_{Tem},X_{Hum}∼N(\theta_{Fre},\nu_{Fre}).$$

Usually, the relationship between each explanatory variable and the response variable should be solved at first. The abovementioned linking mechanism can be defined as generalized linear and non-linear models [26,44], of which the first one has a simpler structure and can be adopted by a wide range of problems. In this study, we establish the linear regression model through the mean response variable given the explanatory variables. It is expressed as

(21) $$Fre=\beta_{0}+\beta_{Tem}X_{Tem}+\beta_{Hum}X_{Hum}+\varepsilon ,$$

where $\beta =[\beta_{0},\beta_{Tem},\beta_{Hum}]$ are the regression parameters for the linear regression model; ε is an error term which satisfies $\varepsilon ∼N(0,\nu_{Fre})$. In other words, the mean of the statistical model can be summarized as

(22) $$E(Fre|X_{Tem},X_{Hum})=\theta_{Fre}=\beta_{0}+\beta_{Tem}X_{Tem}+\beta_{Hum}X_{Hum}.$$

For the response values $\mathrm{Fre}={\left[Fre_{1},Fre_{2},\ldots ,Fre_{n}\right]}^{T}$ and explanatory variable values $X_{i}=[X_{Tem,i},X_{Hum,i}]$ of individuals i=1,2,…,n from the monitoring experiment, the likelihood relationship is written as

(23) $$Fre_{i}∼N(\theta_{Fre,i},\nu_{Fre,i}),$$

(24) $$\theta_{Fre,i}=\beta_{0}+\beta_{Tem}X_{Tem,i}+\beta_{Hum}X_{Hum,i}.$$

As for the SHM information in this paper, the relationships between modal frequencies and environmental factors are firstly established, which are selected as the response and explanatory variables, through linear regression model. The model can be described as

(25) $$Fre|X_{Tem},X_{Hum}∼N(\theta_{Fre}(\beta ,X_{Tem},X_{Hum}),\nu_{Fre}).$$

### 3.2. Dynamic Bayesian Network

**Figure 2:**
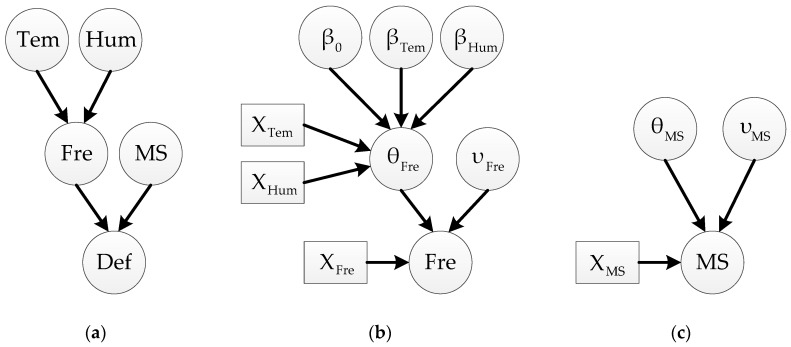
Static BN models: (**a**) Basic BN of deflection analysis with considering main influence factors; (**b**) BN of modal frequency; (**c**) BN of mode shape.

For the overall deflection reliability evaluation with inspection and monitoring data, the linear regression model is not appropriate any more, but BN method can be used to solve this problem. Bayesian network is a kind of directed acyclic graph (DAG), in which the nodes represent the random variables and the arrows depict the relationships among the random variables. The simplified BN for the variables mentioned in this paper is a three-level hierarchical model (shown in Figure 2a), of which the first level is environment factors, the second level is modal parameters and the last one is deflection. Nodes *Tem* and *Hum* represent environmental temperature and humidity, respectively. Nodes *Fre* and *MS* are modal frequency and MS variables, respectively. Node *Def* is the corresponding deflection variable. The first and second levels are connected through the aforementioned linear regression model, which is employed as part of the BN model and depicted particularly in Figure 2b. The residual part of the second level is shown as Figure 2c. The third level can be calculated by the second level through Equation (6). In Figure 2, the circlular and rectangular nodes represent random variables and observation data, respectively.

Generally, if there are a set of variables $X=[X_{1},X_{2},\ldots ,X_{n}]$ in BN model, we can use $Pa(X_{i})$ to denote the parent nodes of node $X_{i}$. According to the basic BN theory, every node $X_{i}$ with the same parent node $Pa(X_{i})$ is conditionally independent from the non-descendant node $X_{j}$ of node $X_{i}$. The general joint probability distribution of these variables [23] is defined as Equation (26) and more details can be seen in Ref. [45].

(26) $$P(X_{1},X_{2},\ldots ,X_{n})=\prod_{i=1}^{n}P(X_{i}|Pa(X_{i})).$$

Here, we take the simplified BN as an example to show the expression of the joint probability distribution.

(27) P(Def,Fre,MS,Tem,Hum)=P(Def|Fre,MS)P(Fre|Tem,Hum)P(MS)P(Tem)P(Hum).

It should be noticed that the variables mentioned above are not static but varying with time. Therefore, DBN are introduced and applied to estimate the dynamic reliability with considering the time-variant properties. Due to the complexity of DBN, we should make some assumptions to simplify the model [46]. Firstly, it is assumed that the conditional probability change process in limited time is uniformly stable for any time slice *t*. Secondly, the dynamic statistical process is satisfied with the property of Markov Chain, namely, the probability at Time *t* + 1 is not dependent with the previous time except the current Time *t*, which is described as Equation (28). Finally, the conditional probabilities of adjacent time are homogeneous, where the transition probability $P(X_{t+1}|X_{t})$ does not depend on time *t*.

(28) $$P(X_{t+1}|X_{0},X_{1},X_{2},\ldots ,X_{t})=P(X_{t+1}|X_{t}).$$

Based on the aforementioned assumptions, DBN can be divided into two portions. One is prior network $N_{0}$ that defines the probability distribution $P_{N_{0}}(X_{0})$ of initial BN, and the other is transition network $N_{\to }$ that expresses the transition probability $P_{N_{\to }}(X_{t+1}|X_{t})$ at any time *t*. Consequently, the joint probabilities of all time slices are described as Equation (29) for the overall DBN defined as $\left(N_{0},N_{\to }\right)$.

(29) $$P(X_{0},X_{1},X_{2},\ldots ,X_{T})=P_{N_{0}}(X_{0})\prod_{t=0}^{T}P_{N_{\to }}(X_{t+1}|X_{t}),$$

where $X_{t}$ represents the variable set at Time *t*.

To be specific, $P_{N_{\to }}(X_{t+1}|X_{t})$ can be extended by the elements of variable set, which is shown as

(30) $$P_{N_{\to }}(X_{t+1}|X_{t})=\prod_{i=1}^{N}P_{N_{\to }}(X_{i,t+1}|Pa(X_{i,t+1})),$$

where $X_{i,t+1}$ is the *i*th variable of $X_{t+1}$ at time slice *t*+1.

So we can obtain the joint probability of arbitrary nodes in DBN model.

(31) $$P(X_{1:N,0:T})=\prod_{i=1}^{N}P_{N_{0}}(X_{i,0}|Pa(X_{i,0}))\prod_{t=1}^{T}\prod_{i=1}^{N}P_{N_{\to }}(X_{i,t}|Pa(X_{i,t})).$$

**Figure 3:**
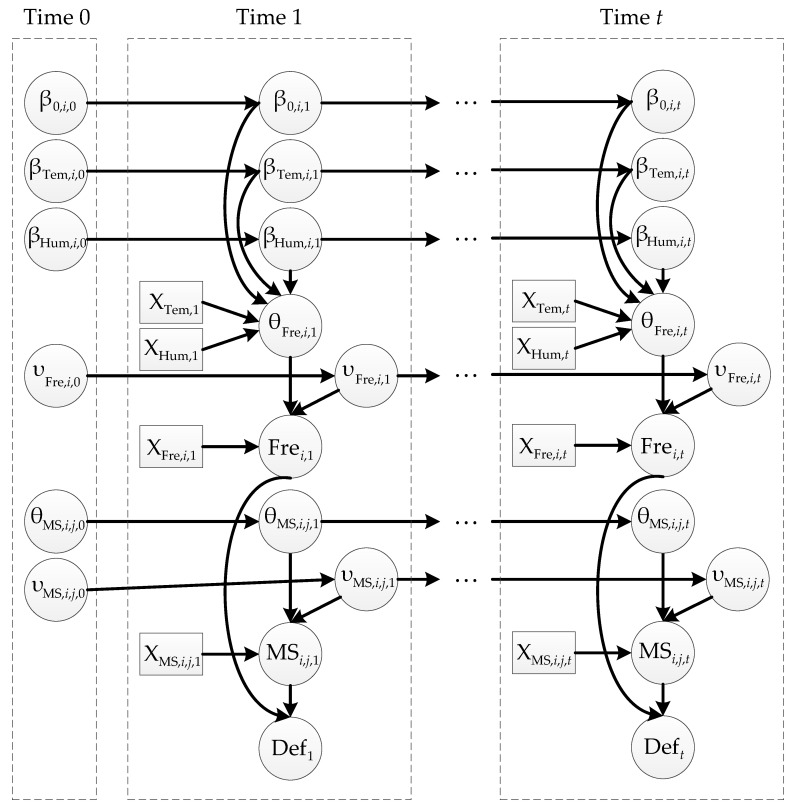
DBN for bridge deflection analysis.

On the basic of the proposed BN model and aforementioned DBN theory, we establish the whole DBN model (depicted in Figure 3) with regard to the dynamic reliability assessment and relevant factors. Each dashed box expresses a time slice and time slice 0 is the prior network $N_{0}$. Any adjacent time slices are composed as the transition network $N_{\to }$. The nodes of the DBN have the same meanings of those in Figure 2b,c. It is worth noticing that the subscripts *i*, *t* representing the *i*th mode at time *t*, and *j* is used to describe the *j*th node of bridge of the *i*th mode at Time *t*. In this study, we monitored the first three modal parameters of the simply supported slab bridge, which is divided into 11 nodes, so *j* = [1, 2, …, 11].

### 3.3. Markov Chain Monte Carlo (MCMC) Simulaiton Method: Gibbs Sampler

**Figure 4:**
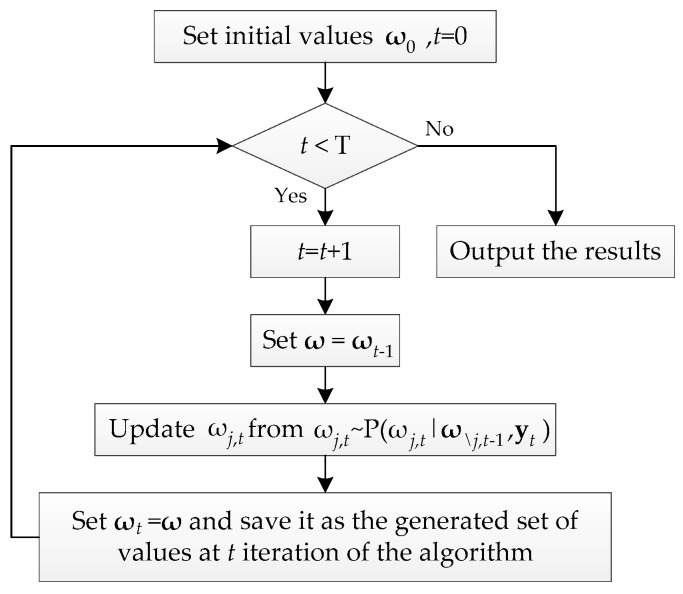
Gibbs sampler algorithm.

Usually, there are two kinds of inference algorithms to solve DBN problem, namely, exact and approximate inference algorithms. The approximate inference algorithm can be suitable for most of complex DBN models while the exact one cannot, which makes the approximate algorithm become a common method. The most popular approximate algorithm is the Metropolis–Hastings algorithm belonging to the family of MCMC simulation methods [43]. Gibbs sampler as a special case of Metropolis–Hastings algorithm is particularly effective because it can exploit the conditional independence properties of DBN [22]. The main steps of Gibbs sampler algorithm is summarized as a flowchart in Figure 4. With the DBN structure and observation data known, the DBN model in this paper can be sampled and solved easily by WinBUGS software [43,47], which is developed based on Gibbs sampler algorithm and used to generate a random sample from the posterior distribution of Bayesian model.

## 4. Experiment Description

### 4.1. Simply Supported RC Slab

**Figure 5:**
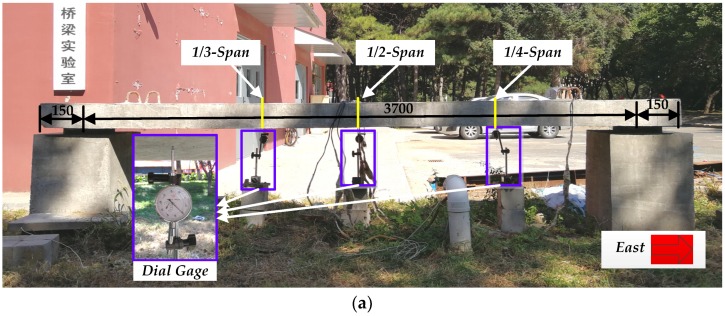

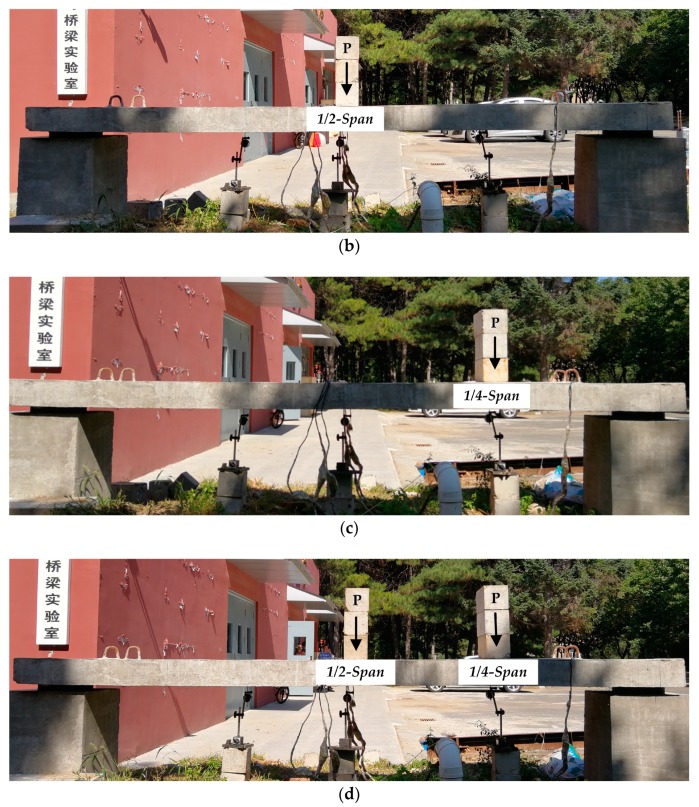
Static test configuration: (**a**) Static test equipment; (**b**) Case 1; (**c**) Case 2; and (**d**) Case 3.

In order to verify the correctness and accuracy of the proposed method, a simply supported slab bridge is used as research object. The deflections at different cases have been measured through static and dynamic tests, respectively. The reinforced concrete (RC) slab (shown in Figure 5) was built on 8 June 2015 with cross section dimensions 600 mm wide and 150 mm high. The length and calculated span length of the slab are 4000 mm and 3700 mm, respectively. There is a 150 mm overhang at each end of the slab. We select common Portland cement of grade 42.5 as cementitious material. Natural river sand with fineness modulus 2.7 and crushed gravels with a nominal maximum size of 31.5 mm are adopted as fine and coarse aggregates, respectively. The mixture proportions are listed in Table 1. To consider the effect of environment on the mass of RC slab, we produced a concrete block under the same condition of the slab to measure the mass variations, which has the same cross section of the slab and length is 400 mm. It was hoisted and placed at supports on 12 September 2015 after cured through pouring concrete in the outside environment for more than 90 days.

### 4.2. Static Test

The static tests were arranged as confirmatory test to verify the correctness of the bridge deflection calculation method proposed in this paper and were not carried out during the period of monitoring. The measure point deflection of simply supported slab bridge was measured by dial gage, of which the precision is 10^−2^ mm. Three dial gages (shown in Figure 5a) were placed at 1/4, 1/2, and 1/3 of the calculated span length from east to west, which were defined as 1/4-Span, 1/2-Span, and 1/3-Span, respectively. Three loading configurations were designed and implemented as shown in Figure 5b,d, namely, Case 1 with a single load located at 1/2-Span, Case 2 with a single load located at 1/4-Span and Case 3 with two single loads located at 1/2-Span and 1/4-Span. Mass of a single load is 48 kg, in other words, load P is 470.4 N. Every case was measured four times for both the static and dynamic tests of confirmatory test so as to avoid the effect of test errors.

### 4.3. Bridge Structural Health Monitoring Process

#### 4.3.1. Dynamic Test

**Figure 6:**
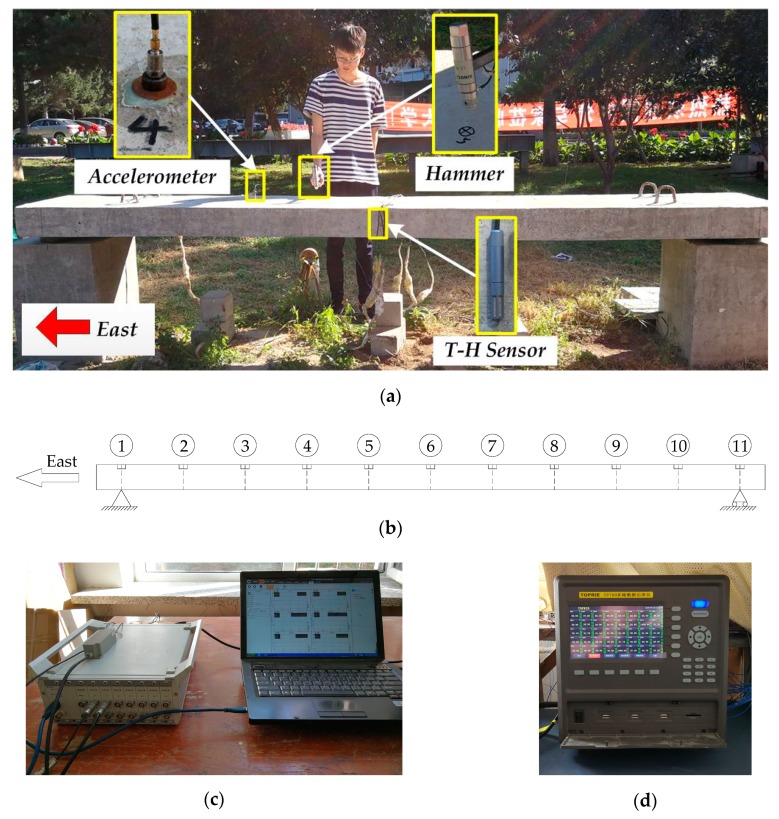
Structural health monitoring system configuration: (**a**) monitoring sensors; (**b**) node numbers; (**c**) DH5922 type dynamic test system; and (**d**) TP700 multichannel data recorder.

In order to obtain modal parameters accurately, a DH5922 type dynamic signal measurement and analysis system (shown in Figure 6c) is used to measure and analyze modal frequency and MS. It is manufactured by Donghua Testing Technology Corporation in China and includes 16 24-bit integrated electronics piezoelectric (IEPE) input channels. We place the DH5922 system in laboratory on account of its operating temperature ranging from 0 to 40 °C. This dynamic test system includes two test patterns for mode analysis, namely, force measurement method and non-force measurement method. The former method is implemented by an accelerometer and an impulse hammer with a piezoelectric force sensor, while the latter one is carried out with accelerometers arranged at every node. The force measurement method has the better performance and the higher accuracy, whereas the other one is widely used in actual engineering due to its easy implementation. In this study, we choose the force measurement method for mode analysis.

DH131E accelerometers produced by the same manufacturer of DH5922 system are selected as the vibration sensors. The accelerometer features a sensitivity of 1 mV/g and has the advantages of large frequency range (1–8000 Hz), small size (φ10 × 16 mm) and light weight (5.5 g). The operating temperature of DH131E accelerometer ranges from −40 to 80 °C, which can be applied in outside environment. An impulse hammer manufactured by Wuxi Yutian Technology Corporation is used in this test and it features a pressure range of 0–60 kN and a sensitivity of 4.12 pC/N. Its operating temperature ranging from −50 to 150 °C makes it ideal for outdoor application. The accelerometer and impulse hammer are depicted in Figure 6a.

Eleven measurement points are arranged on the slab. Two measurement points are located at the supporting points and the residual points are distributed uniformly between two supporting points. In other words, there are eleven lumped masses in the spatial model. These points are numbered successively as node 1 to node 11 from east supporting point to the west one, which is illustrated in Figure 6b. The accelerometer installed at node 4 is mounted on the magnetic base fixed on the upper surface of slab by epoxy resin adhesive. Both the accelerometer and the impulse hammer are connected with the DH5922 system using L5 coaxial extension cables with lengths of 15 m. The frequency response function excited by the impulse hammer is measured for each of the measurement points. Modal parameters can be obtained using DHDAS software with the sampling frequency set to be 1000 Hz and the frequency resolution set as 0.313 Hz.

#### 4.3.2. Environment Temperature and Humidity Monitoring System

A temperature and humidity monitoring system was designed to measure the real-time air temperature and humidity of the environment, where the slab was located. This system consisted of a TP-2307 digital temperature and humidity (T&H) sensor and a TP700 multichannel data recorder including T&H input module (shown as Figure 6d), and they were both manufactured by TOPRIE Electronics Corporation in China. The T&H sensor can measure the air temperature ranging from −40 to 125 °C with 0.3 °C temperature resolution and the air relative humidity ranging from 0% to 99% with 0.3% humidity resolution. It was fixed on the undersurface of the slab, which is shown in Figure 6a. To ensure the measurement accuracy, the TP700 recorder was installed in laboratory because of its operating temperature ranging from 0 to 50 °C.

The monitoring tests were implemented from 8 October 2015 to 25 September 2016 and the actual monitoring time was 62 days. Modal testings with three parallel tests were carried out every two hours from 8:00 a.m. to 10:00 p.m. in the days of monitoring period. In total, 1488 modal parameter monitoring tests were conducted. At one-minute intervals during the same time, air temperature and humidity were measured and recorded by the temperature and humidity monitoring system. There are slight changes for the environment parameters from 10:00 p.m. to 8:00 a.m. the next day, and Mosavi et al. [48] demonstrated that the first five modal parameters did not change obviously between night and morning measurements. We could not perform the monitoring process continuously due to the restrictions of equipment. In addition, the mass block was weighed at the same time of monitoring tests.

## 5. Results and Discussions

### 5.1. Validation of Deflection Calculation

The comparison between the static and dynamic test results under different cases was performed to validate the correctness and efficiency of the bridge deflection calculation method based on the modal theory and the improved Kriging method. The deflection results of three measure points are described in Table 2. For each case, the deflections at 1/4-Span, 1/2-Span, and 1/3-Span were all measured four times. Although the deflections are different for the same measure point every time, both the absolute difference value between any two test results and the coefficient of variant (COV) are so small that we have reasons to believe the real deflection can be obtained by the static test method. The mean deflections are chosen to be compared with the values measured through the proposed dynamic method. As can be seen in Table 2, the largest mean deflection of every cases all occurs at 1/2-Span, namely the middle span of bridge. Those values at 1/3-Span are larger than those at 1/4-Span except Case 2, of which the external force is loaded on 1/4-Span. Because Case 3 can be regarded as a case composed by Cases 1 and 2, the result reveals that the sum of deflections at the same measure point in Cases 1 and 2 equals to that of Case 3 approximately, which is consistent with the superposition principle.

**Figure 7:**
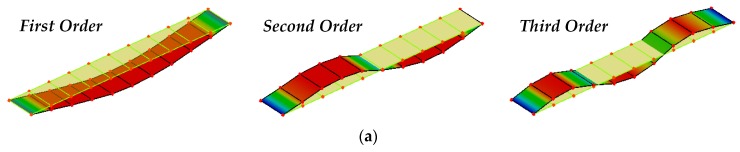

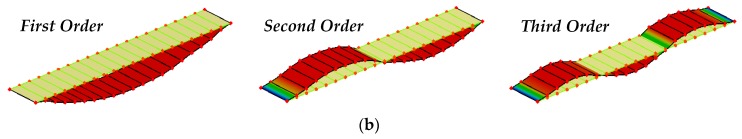
The first three vertical MSs of the bridge: (**a**) 11 nodes; (**b**) 21 nodes.

As for the deflection calculated through the dynamic method, we carried out the modal testing with the bridge divided into 11 nodes and 21 nodes, respectively, and extracted the first three vertical vibration modal parameters by the DH5922 type dynamic signal measurement and analysis system. Figure 7 illustrates the first three vertical MSs analyzed by DHDAS-2013 software platform which is an important part of the DH5922 system.

If the bridge is divided into more nodes, deflections at more nodes can be acquired more preciously. However, the modal analysis with more nodes will make it time-consuming and uneconomically. The proposed deflection calculation method based on modal flexibility and improved Kriging method can solve this problem perfectly, even though MSs with just a few nodes are obtained. Here the mode shapes with 21 nodes are chosen as the benchmark ones, and the ones of 11 elements as the input information are utilized to calculate the mode shapes of 21 elements through the surrogate model. The Kriging method is constructed with regression model and correlation model. The regression models are abbreviated as RM0, RM1, and RM2 for zero, first and second order polynomials in this paper, respectively. Gaussian model has been widely used as a common correlation model due to its good applicability. Besides, the parameter θ in correlation model is another essential part which controls the performance of Kriging model. The Kriging method is implemented in the toolbox DACE of MATLAB [37].

**Figure 8:**
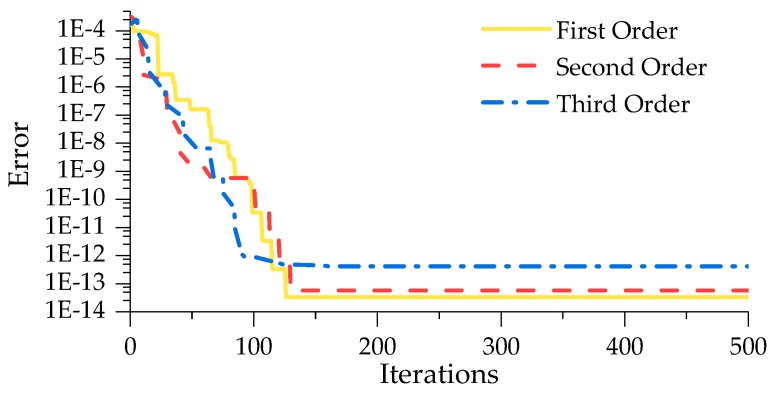
The iteration process for searching the best θ.

**Figure 9:**
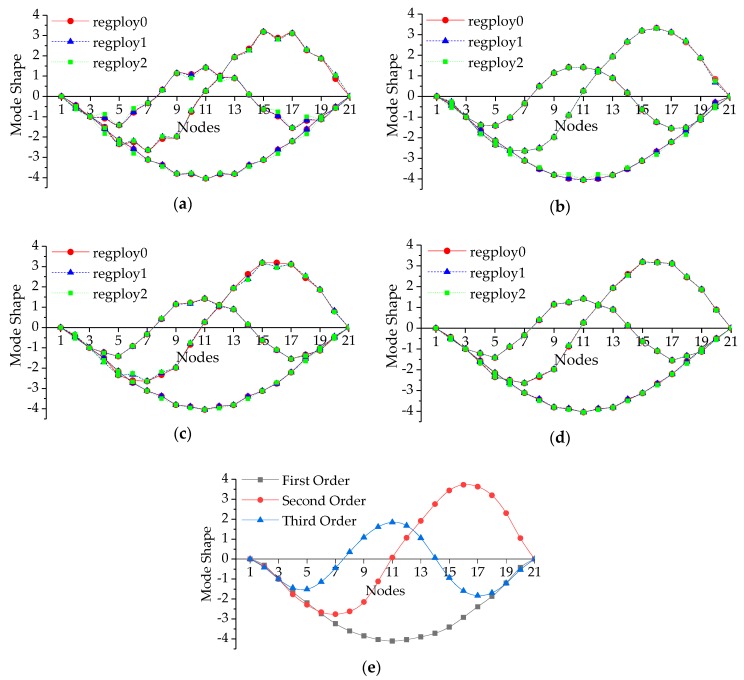
The first three vertical mode shapes simulated by different Kriging models and measured actually: (**a**) exponential model; (**b**) gaussian model; (**c**) linear model; (**d**) spherical model; (**e**) actual measured MSs.

In this article, we establish the optimal surrogate model by the improved Kriging method based on ABC algorithm, of which the particular process has been depicted in Figure 1. As for the parameter values of ABC, we refer the literatures [40,41] and the bee colony size, the control parameter and the maximum number of iterations are set to be 50, 50, and 500, respectively. The object function of ABC algorithm is defined by a Euclidean distance between the *i*th order simulated and measured MSs (expressed as Equation (32)), which is also used as an error index to evaluate the fitting quality. It should be noticed that *N* equals to 11 at this time. The corresponding iteration processes for searching the best θ for the first three modes are shown in Figure 8. Therefore, we can obtain an improved Kriging model using the best θ. Due to the influence of regression and correlation models, we determine the optimal combination of them with comparing the simulated and measured results depicted in Figure 9 which illustrates the simulated first three vertical MSs with 21 nodes. Once more, the index defined by Equation (32) is employed to estimate the error (described in Table 3) between the actual measured and simulated MSs with 21 nodes.

(32) $$Error_{i}=\sqrt{\sum_{j=1}^{N}{\left(M_{Si,j}-M_{Mi,j}\right)}^{2}},$$

where $Error_{i}$ is the error between the *i*th order simulated and measured MSs; $M_{Si,j}$ is the *j*th node values of the *i*th order simulated MS vector while $M_{Mi,j}$ is that of the actual measured one; *N* is the number of nodes.

As can been seen in Figure 8, ABC algorithm converges at 130 iterations approximately, and the magnitudes of error decreases from 3.15 × 10^−4^ to 3.36 × 10^−14^. Here, just a typical set of the iteration processes is presented in Figure 8 and the maximum error for all the iteration process results does not exceed 1 × 10^−12^. It demonstrates that the improved surrogate model has favorable applicability and can identify the object MSs accurately. It is a remarkable fact that the Kriging method composed by different combination of regression and correlation models with its corresponding best parameter θ, has sufficient accuracy to fit MSs.

Through the comparison in Figure 9, we can confirm the surrogate model constructed with the RM0 or RM1 and Gaussian models has the best performance for MSs fitting intuitively. The simulated MSs in Figure 9b, which are visually same with the actual measured ones in Figure 9e, are smoother than those in Figure 9a,c,d. According to Table 3, the Kriging method based on Gaussian model possesses the smallest error except combining with RM2 for first order MSs, while that based on the exponential model possesses the largest error. As for the method composed by the other two correlation models, its error ranges from 0.3532 to 0.7229, which proves they cannot simulate the MSs precisely. From the perspective of the regression models combined with Gaussian model, the errors caused by RM0 and RM1 are approximate, and RM2 provides larger error. In addition, it is worth mentioning that there is a huge gap between the errors shown in Figure 9 and Table 3, which represent the errors between the actual measured MSs with 11 or 21 nodes and the corresponding ones simulated by the Kriging model based on the actual MSs with 11 nodes, respectively. The error in Figure 9 is caused by the Kriging method, while that in Table 3 is induced by the error between two testing results besides the method itself. Therefore, this phenomenon demonstrates that the improved Kriging method can simulate the existing testing data precisely and possesses the ability to predict any unknown node values.

In conclusion, the improved Kriging model composed with the RM0 and Gaussian models performs better than the others, and possesses the smallest error (no more than 0.12). Based on the optimal surrogate model, MSs with all external forces located at nodes can be generated, even when there are only a few MS nodes in the dynamic testing results.

**Figure 10:**
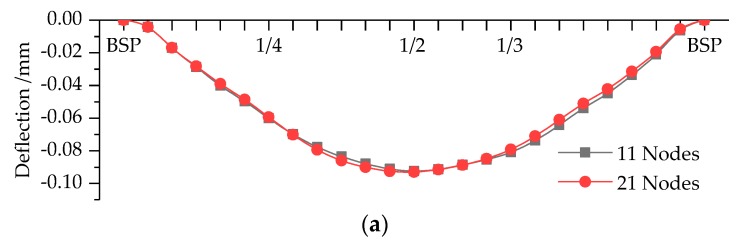

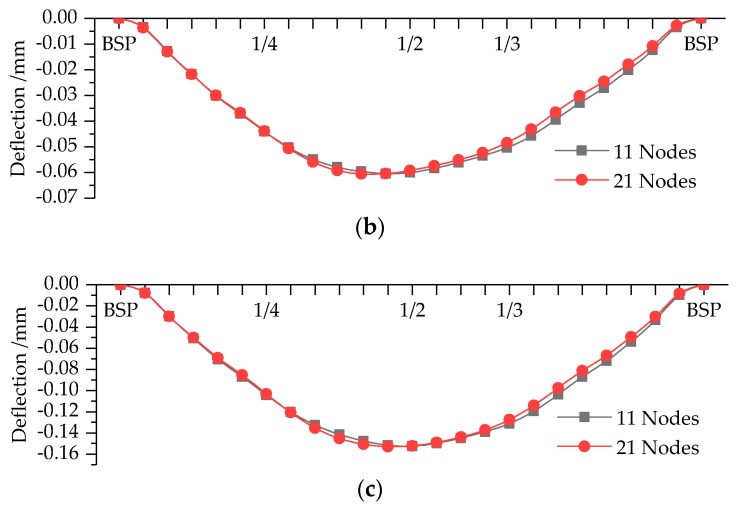
Bridge deflection curves modeled by Kriging method: (**a**) Case 1; (**b**) Case 2; and (**c**) Case 3.

In the second stage of the proposed bridge deflection calculation method, if the deflection of target position cannot be computed through the simulated MSs directly, it will be achieved by employing the improved Kriging method again. Here the method composed with RM0 and Gaussian model also perform the best for this part, and its iteration process for ABC algorithm and the fitting accuracy are similar to the aforementioned results. Bridge deflection curves modeled by Kriging method are depicted in Figure 10, of which BSP, 1/4, 1/2, and 1/3 represent bridge support point, 1/4-Span, 1/2-Span, and 1/3-Span, respectively. Table 4 illustrates the particular deflections calculated by the proposed method based on modal flexibility and the improved Kriging method, which are compared with the static results in order to validate the proposed method.

As shown in Table 4, we also carried out four times dynamic testing for both MSs with 11 and 21 nodes and calculated the deflections at measure points of Cases 1 to 3. The COVs of deflection results are less than 0.0427, which demonstrates the proposed method can compute deflection steadily. There are not obvious differences between the results based on MSs with 11 and 21 nodes. In order to terrify the correctness of the proposed method, we utilize relative error between the mean deflections calculated by static and dynamic method as the assessment index. We can confirm that the absolute values of relative error do not exceed 5% except the results at 1/4-Span of Cases 2 and 3 computed by mode shapes with 11 nodes, and the absolute relative error for 11 nodes are generally lager than those for 21 nodes under the same cases. The reason lies in that the deflections are calculated through the improved Kriging method of two times, which inevitably leads to expand errors. There is a certain difference between the predicted and actual measured MSs as described in Table 3. In addition, 1/4-Span is close to support point, so the accuracy of testing result will be influenced easily. As for the other measure points, the relative error indicates the proposed method can calculate the deflection successfully. The only drawback of the proposed method is that it cannot precisely compute deflection at measure point which is close to support point. Therefore, we have reasons to believe the proposed method based on modal flexibility and improved Kriging model can calculate the deflection of simply supported bridge correctly and precisely.

In this paper, we only take a simple supported bridge as a research object and verify the correctness and accuracy of the proposed method. However, the proposed method for bridge deflection calculation can also be applicable for other bridge types such as concrete continuous box girder bridge [19] and irregular bridge. As bridge age increases, bridge damage is inevitable. When bridge structure is damaged, bridge deflection curve will be changed. Modal parameters depend on the state of bridge and modal flexibility has been widely used as an indicator for bridge damage identification. Bridge deflection under damage condition can be calculated by the proposed method in this paper. In other words, bridge damage is another influence factor for deflection calculation. In the future studies, we will investigate and verify the applicability of the proposed bridge deflection calculation method for damaged structure and other bridge types.

### 5.2. DBN Result Analysis

**Figure 11:**
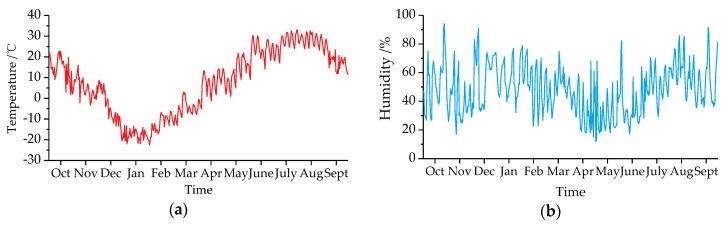
Environment parameters variations with months: (**a**) temperature; (**b**) humidity.

**Figure 12:**
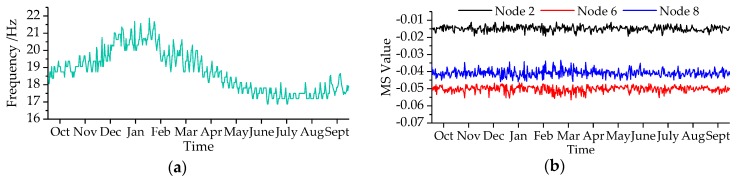

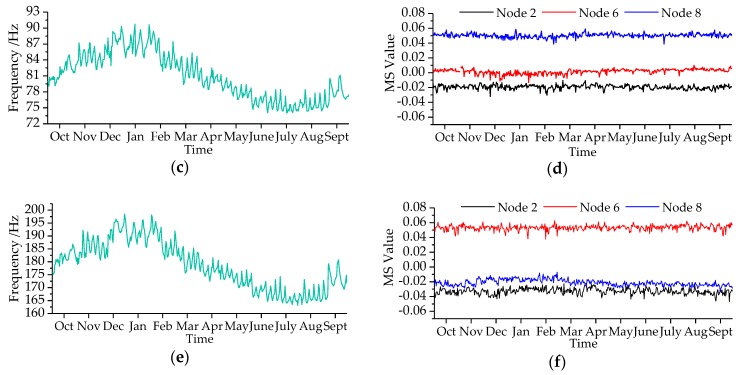
Modal parameters variations with months: (**a**) first order frequency; (**b**) first order MS; (**c**) second order frequency; (**d**) second order MS; (**e**) third order frequency; (**f**) third order MS.

Through the bridge SHM system, the environment temperature and humidity were measured and shown in Figure 11. It revealed environment temperature of cold region varied widely, which ranged from −22.5 to 32.5 °C affected by an annual cycle. However, no obvious rule for relative humidity variation was observed. Figure 12 illustrates the variations of the first three modal parameters with time. The first three modal frequencies have opposite trends with temperature variations. In the monitoring process, we identified maximum displacement normalized MS with 11 nodes and transformed it into MNMS by Equation (4). Nodes 2, 6, and 8 were selected as the typical ones to demonstrate the MS value variations, which revealed that there were little changes in the MS.

With the observed SHM data, WinBUGS was utilized to analyze the DBN depicted in Figure 3, of which the iterations were set to be 6000, and the initial 1000 ones were discarded as burn-in. The monitored data in a month was used for a time slice of DBN, namely Time 1 to Time 12 were corresponded with October 2015 to September 2016. In this paper, we assumed the regression coefficient variable β and mean variable θ following normal distribution, and variance variable ν following gamma distribution, because normal and gamma distribution were demonstrated to be appropriate for mean and variance variables in reference [43]. It should be noticed that if the prior information for Time 1 is unknown, it can be regarded as low-information prior. Therefore, we set prior mean and variance as 0 and 10^4^ to represent high uncertainty. A part of posterior summaries of first order in Time 1 are selected as typical results and described in Table 5.

Here, MC error is used to reflect the estimated accuracy of posterior information. If MC error is lower than 1/20 of its corresponding posterior standard deviation, we can assume DBN converges with high precision. Not limited to the posterior results in Table 5, all the variables are satisfied with the convergence criteria. Through Kolmogorov–Smirnov test, it demonstrates posterior distribution of variables follow the same probability distribution with their priors. Based on the posterior means, we can end up with regression model of frequency. The regression models for the first three order frequencies at Time 1 are written as

(33) $$Fre_{1,1}=19.04-0.02911\times X_{Tem}-0.006749\times X_{Hum},$$

(34) $$Fre_{2,1}=84.66-0.1705\times X_{Tem}-0.02288\times X_{Hum},$$

(35) $$Fre_{3,1}=186-0.3493\times X_{Tem}-0.0535\times X_{Hum}.$$

As can be seen from Equations (33)–(35) and Table 5, it can be inferred that both environment temperature and humidity have important effects on modal frequencies. For every increase of the temperature by 1 °C, the decrease of first modal frequency at Time 1 lies between 0.03546 Hz and 0.02273 Hz with probability 95%. This phenomenon is consistent with the conclusion in reference [29]. When humidity increases 1%, the frequency is expected to decrease 0.006749 Hz averagely. According to modal frequency calculation formula of simply supported bridge, modal frequency will decrease when bridge mass increase. The increase of humidity may cause a little of increase of bridge mass, so it coincides with the modal theory. With mode order increasing, modal frequency values will be influenced by these two environment factors more obviously. The variation intervals of the third modal frequency even reach up to 0.3493 Hz and 0.0535 Hz on average for unit change of temperature and humidity, respectively. Considering another perspective, it also demonstrates that humidity has much less contribution on modal frequency variations than temperature. The total variations of the first three modal frequencies influenced by humidity are 0.493 Hz, 1.67 Hz, and 3.91 Hz. They are all lager than the frequency resolution, which illustrates the observed influence of humidity is not caused by measurement noise. The standard deviations of all variables are so small that the posterior distributions of modal frequencies and MSs can be predicted precisely through the posterior parameters shown in Table 5. The variables not listed in Table 5 have the same variation trends and rules. In addition, it should be noticed that the test environment belongs to cold region, which means the results only can be referred for bridges in cold region. However, the proposed method can be used for any kind of environments.

### 5.3. Dynamic Reliability Analysis of Simply Supported Bridge Deflection

As described in the sections mentioned above, we proposed the simply supported bridge deflection calculation method and elaborated the DBN analysis process for probability distributions of relative modal parameters. According to the standard requirements of design code [49], maximum deflection value, which generally locates at 1/2 span of bridge, cannot be more than 1/250 of its span length *l* when *l* is less 7 m. Hence, the performance function for simply supported bridge deflection can be defined as Equation (36), in which the parameter meanings are identical to those of Equations (5) and (6).

(36) $$Z=\frac{l}{250}-\sum_{i=1}^{r}f_{j}\frac{1}{\omega_{i}^{2}}{\bar{\varphi }}_{j,i}{\bar{\varphi }}_{i}^{T}.$$

The corresponding failure probability (FP) and reliability index (RI) can be defined as

(37) FP=Pr(Z<0),

(38) $$RI=-\Phi^{-1}[\mathrm{Pr}(Z<0)],$$

where $\Phi^{-1}$ represents the inverse standard normal cumulative distribution function.

**Figure 13:**
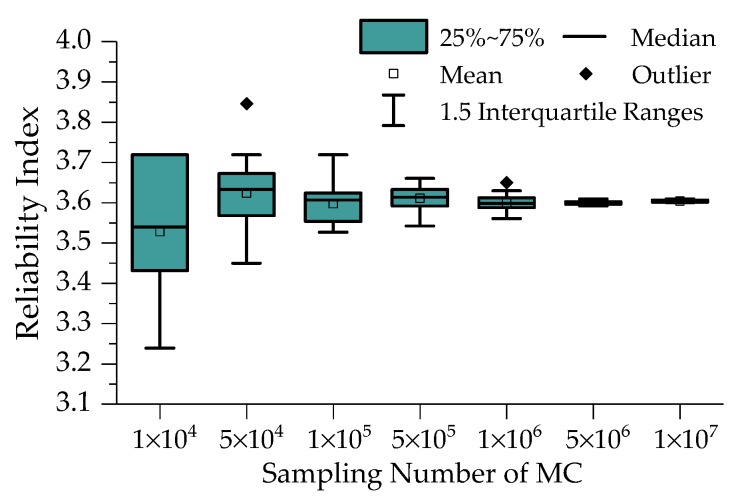
Boxplot of RI with different sampling numbers.

In this paper, the performance function is calculated based on modal flexibility and Kriging method, which is difficult to obtain the closed-form solution of Equation (37). So Monte Carlo (MC) simulation method is employed to solve this problem. MC method possesses the best performance in calculation accuracy and applicability except computation time, which increases with the sampling number. Based on DBN analysis results of the variables probability distribution, we take the RI calculation process of Case 1 in January 2016 with 20 kN external force as an example. The sampling numbers are set to be 1 × 10^4^, 5 × 10^4^, 1 × 10^5^, 5 × 10^5^, 1 × 10^6^, 5 × 10^6^, and 1 × 10^7^, and it is computed twenty times for each sampling number. The boxplot of computation results is depicted in Figure 13. With sampling number increasing, the dispersion of RI results decreases gradually, and it converges when the sampling number exceeds 1 × 10^6^. The computation time of MC method with sampling number 5 × 10^6^ is less than 2 min, while that for 1 × 10^7^ is even more than 15 min. The former is within the acceptable range, so the sampling number for MC method is set to be 5 × 10^6^ in the following computations.

**Figure 14:**
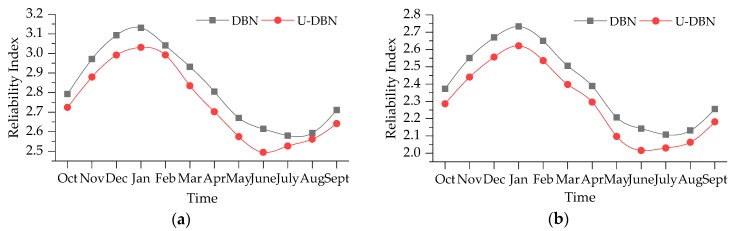
Time-dependent RI: (**a**) Case 1; (**b**) Case 3.

In order to analyze the dynamic reliability of simply supported bridge, we calculated the reliabilities of Case 1 and Case 3 mentioned above, respectively. The external forces for these two cases are both assumed lognormal distributed with mean 15 kN and standard deviation 6 kN. The time-dependent reliabilities based on DBN method are shown in Figure 14. They are compared with that calculated through the probability distribution (U-DBN), which are identified by the monitoring data from October 2015 to September 2016.

In Figure 14, it is shown that the time-dependent reliability is negatively correlated with environment temperature (shown in Figure 11a). The maximum RIs of Cases 1 and 3 calculated by DBN method are 3.13 and 2.73, while the minimum ones are 2.58 and 2.11. For Cases 1 and 3, the minimum RIs decreases by 17.6% and 22.7% compared with the maximum ones. The maximum and minimum RIs are corresponding to January and July, which have the lowest and highest temperature, respectively. We can also confirm that the RIs calculated through U-DBN are all lower than the results of DBN, namely it underestimates the bridge deflection reliability. According to the correlation between the reliability and temperature, the lowest RI should occur in July; however, the lowest RI calculated by U-DBN occurs in June in Figure 14. It demonstrates the proposed bridge deflection reliability calculation method based on DBN can reduce the influence of uncertainty and improve the reliability assessment accuracy.

**Figure 15:**
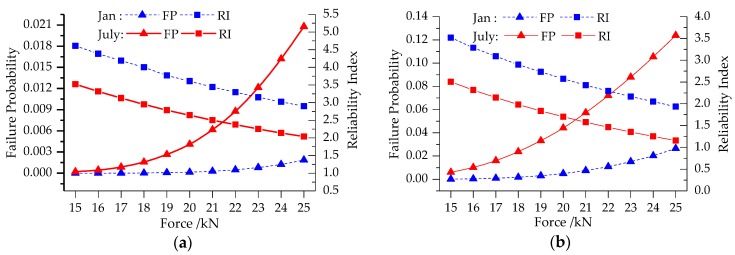
RI with external force variation: (**a**) Case 1; (**b**) Case 3.

Figure 15 depicts the bridge deflection FPs and RIs of January and July with external forces from 15 to 25 kN. The RIs of January range from 4.61 to 2.90 for Case 1 and from 3.52 to 2.04 for Case 3, while those of July range from 3.52 to 1.93 for Case 1 and from 2.50 to 1.16 for Case 3. The FP curves present exponential with external force increasing. RI can be utilized to determine vehicle load limitation of actual bridge. In actual engineering, vehicle load limitation is set according to ultimate limit states of bridge without considering environment influence and structural health monitoring, and it seems to be not safe or accurate enough. However, it can be provided reasonably through the reliability calculation method proposed in this paper. For example, if the target RI is set as 3.0, the external force should be limited lower than 17 kN for Case 1.

### 5.4. Sensitivity Analysis of the Variables

**Figure 16:**
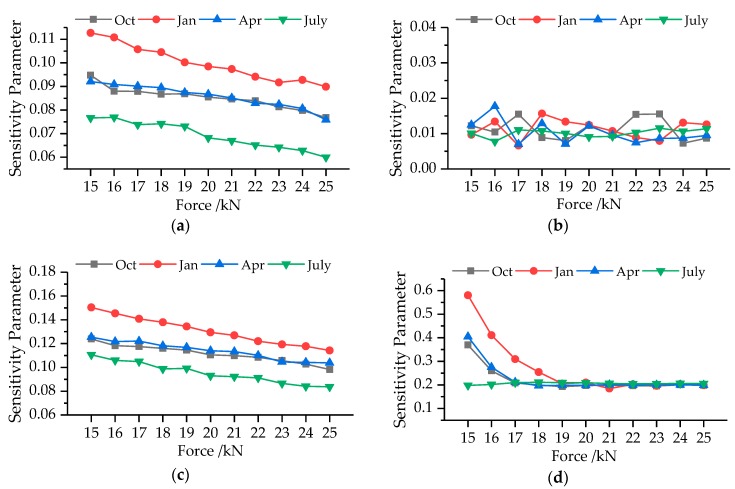
SP for the influence factors with external force variation: (**a**) temperature; (**b**) humidity; (**c**) modal frequency; (**d**) MS.

In this work, we computed the dynamic RIs combining with the modal parameters and environment factors. To investigate the effect of influence factors on the RI results, a quantitative sensitivity parameter introduced in reference [50] is employed here (defined as Equation (39)). The RI sensitivity results of modal frequency, MS, temperature and humidity in October 2015, and January, April, and July 2016 for Case 1 are shown in Figure 16.

(39) $$SP=\frac{RI_{IF}-RI}{RI},$$

where SP represents the quantitative sensitivity parameter; RI is the original reliability index mentioned above; $RI_{IF}$ is the reliability index calculated without considering one of the influence factors (IF), namely it is taken as a constant while other factors are maintained as statistical variables.

As can be seen from Figure 16, environment temperature, modal frequencies and MSs are major parameters affecting the bridge deflection RI. The SP of environment humidity is approximately 0.01 and ranges between 0.05 and 0.02 irregularly, which reveals humidity has little influence on bridge deflection RI. As for temperature and frequencies, the SP decreases linearly with force increasing, which demonstrates these two factors play less important roles in the RIs when external forces become larger. In Figure 16a,c, the SP in January possesses the largest value while that in July is the smallest under the same condition. SP in April is slightly larger than that in October in most conditions. This phenomenon illustrates that temperature and frequencies have more important effects on bridge deflection RI in low temperature environment. In addition, the largest and smallest values in Figure 16a are 0.113 and 0.059, and those in Figure 16c are 0.151 and 0.084. Modal frequency makes more contribution on bridge deflection RI than temperature. In the SP curves for MS, there is a critical force value. The SP decreases with force increasing when external force is smaller than the critical force value and becomes a constant when external force is larger than the critical force value. The critical force value becomes larger as the environment temperature decreases. Because temperatures in April and October are similar, the SP curves have the same variation tendency. The largest SPs of MS are 0.581, 0.405, 0.369 and 0.211 in January, April, October, and July, while the smallest one is about 0.2 for all of them. Therefore, the bridge deflection RI is more sensitive to MS than the other three factors.

## 6. Conclusions

In this study, a deflection calculation method of simply supported bridge based on modal flexibility theory and the improved Kriging method is proposed. It can be used to monitor bridge deflection through dynamic signal measurement and analysis system with acceleration sensors. We also established an RC bridge used to validate the correctness of the proposed method and obtain the SHM data. Through DBN and the sensitivity parameter, we carried out dynamic bridge deflection reliability analysis. The following conclusions can be drawn

(1) The error between the actual tested MS and the simulated results was reduced from 3.15 × 10^−4^ to 3.36 × 10^−14^ by using the Kriging method improved by ABC algorithm. The improved Kriging model constructed by zero order polynomial and Gaussian model possessed the best performance to predict MS with 21 nodes based on the existing MS with 11 nodes. It demonstrated that the proposed method can calculate bridge deflection accurately comparing with the results measured by the static testing. For all the cases, the relative error was less than 5% except two cases, of which the errors were 7.62% and 5.33%.
(2) The posterior probability distributions of the variables were effectively analyzed by DBN. The linear regression models of the first three order frequencies revealed that both environment temperature and humidity were negatively correlated with the frequencies. Humidity had much less of a contribution than temperature on modal frequency variation. The posterior distribution of MS did not change obviously. The posterior standard deviations were so small that the DBN analysis can reduce the uncertainty of variables.
(3) The bridge deflection time-dependent RIs of Cases 1 and 3 were calculated by MC method. The results illustrated the RI reached maximum in January and minimum in July, and was also negatively correlated with environment temperature. The RIs calculated through U-DBN were not precise enough and underestimated the bridge deflection reliability. Vehicle load limitation can be provided reasonably through the reliability calculation method proposed in this paper.
(4) Through the sensitivity analysis, humidity had little influence on bridge deflection RI. The SP of temperature and modal frequency decreased linearly with force increasing, and had more of an effect on bridge deflection RI in low temperature environments. There was a critical force value in the SP curves for MS. The SP decreased with force increasing when external force was smaller than the critical force value, and became a constant when external force was larger than the critical force value. The bridge deflection RI was more sensitive to MS than the other three factors.

## Figures and Tables

**Table 1:** Mixture proportions of slab.

Material	Unit	Nominal Proportions
Cement	kg/m^3^	378
Coarse aggregate	kg/m^3^	1230
Fine aggregate	kg/m^3^	607
Water	kg/m^3^	185
Water/cement ratio	—	0.49

**Table 2:** Deflection results measured by static test method (unit: mm).

Case Number	Measure Point	Test Times				Mean	COV
		1	2	3	4		
Case 1	1/4-Span	0.06	0.05	0.06	0.07	0.0600	0.1179
	1/2-Span	0.09	0.08	0.09	0.10	0.0900	0.0786
	1/3-Span	0.07	0.07	0.08	0.08	0.0750	0.0667
Case 2	1/4-Span	0.05	0.05	0.06	0.05	0.0525	0.0825
	1/2-Span	0.06	0.06	0.07	0.06	0.0625	0.0693
	1/3-Span	0.05	0.04	0.06	0.05	0.0500	0.1414
Case 3	1/4-Span	0.11	0.12	0.11	0.11	0.1125	0.0385
	1/2-Span	0.14	0.15	0.16	0.15	0.1500	0.0471
	1/3-Span	0.12	0.13	0.12	0.13	0.1250	0.0400

**Table 3:** Errors between actual measured and simulated MSs with 21 nodes.

Correlation Models	Regression Models								
	First Order			Second Order			Third Order		
	RM0	RM1	RM2	RM0	RM1	RM2	RM0	RM1	RM2
Exponential	0.5857	0.5392	0.5722	0.9211	1.0887	1.0985	0.7399	0.7996	1.2130
Gaussian	0.1022	0.1160	0.5705	0.1034	0.1346	0.1750	0.1153	0.1483	0.1744
Linear	0.4213	0.4451	0.3549	0.4525	0.6219	0.7229	0.4048	0.4409	0.3890
Spherical	0.3532	0.3851	0.3796	0.3647	0.4169	0.4150	0.4091	0.4177	0.3930

**Table 4:** Deflection results measured by dynamic test method (unit: mm).

Case Number	Measure Point	Number of Nodes	Test Times				Mean	COV	Relative Error
			1	2	3	4			
Case 1	1/4-Span	11	0.056	0.056	0.059	0.061	0.0580	0.0366	−3.33%
		21	0.060	0.062	0.063	0.060	0.0613	0.0212	2.08%
	1/2-Span	11	0.087	0.090	0.080	0.087	0.0860	0.0427	−4.44%
		21	0.093	0.096	0.092	0.093	0.0935	0.0160	3.89%
	1/3-Span	11	0.075	0.078	0.076	0.079	0.0770	0.0205	2.67%
		21	0.077	0.079	0.072	0.077	0.0763	0.0339	1.67%
Case 2	1/4-Span	11	0.046	0.049	0.050	0.049	0.0485	0.0309	−7.62%
		21	0.049	0.051	0.052	0.050	0.0505	0.0221	−3.81%
	1/2-Span	11	0.059	0.060	0.061	0.059	0.0598	0.0139	−4.40%
		21	0.060	0.062	0.063	0.060	0.0613	0.0212	−2.00%
	1/3-Span	11	0.051	0.052	0.054	0.051	0.0520	0.0236	4.00%
		21	0.047	0.047	0.050	0.049	0.0483	0.0269	−3.50%
Case 3	1/4-Span	11	0.102	0.105	0.109	0.110	0.1065	0.0301	−5.33%
		21	0.109	0.113	0.115	0.110	0.1118	0.0213	−0.67%
	1/2-Span	11	0.146	0.150	0.141	0.146	0.1533	0.0219	−2.83%
		21	0.153	0.158	0.155	0.153	0.1473	0.0132	3.17%
	1/3-Span	11	0.126	0.130	0.130	0.127	0.1290	0.0134	3.20%
		21	0.124	0.126	0.122	0.126	0.1245	0.0133	−0.40%

**Table 5:** Dynamic Bayesian network results in Time 1.

Variables	Mean	Standard Deviation	MC Error	2.5% Value	Median Value	97.5% Value	Distribution
β_0,1,1_	19.04	0.07693	0.001008	18.89	19.04	19.19	normal
β_Tem,1,1_	−0.02911	0.003259	4.54×10^−5^	−0.03546	−0.02908	−0.02273	normal
β_Hum,1,1_	−0.006749	0.001376	1.831×10^−5^	0.004072	0.006756	0.009463	normal
θ_Fre,1,1_	19.06	0.02339	3.433×10^−4^	19.01	19.06	19.1	normal
ν_Fre,1,1_	0.04185	0.007036	8.997×10^−5^	0.03028	0.04122	0.05757	gamma
θ_MS,1,2,1_	−0.01494	0.001846	2.308×10^−5^	−0.01849	−0.01495	−0.01124	normal
ν_MS,1,2,1_	2.72×10^−4^	4.531×10^−5^	5.204×10^−7^	1.975×10^−4^	2.674×10^−4^	3.719×10^−4^	gamma
θ_MS,1,6,1_	−0.05022	0.001848	2.31×10^−5^	−0.05378	−0.05024	−0.04652	normal
ν_MS,1,6,1_	2.726×10^−4^	4.54×10^−5^	5.214×10^−7^	1.979×10^−4^	2.679×10^−4^	3.726×10^−4^	gamma
θ_MS,1,8,1_	−0.04102	0.001852	2.316×10^−5^	−0.04459	−0.04103	−0.03731	normal
ν_MS,1,8,1_	2.739×10^−4^	4.561×10^−5^	5.239×10^−7^	1.989×10^−4^	2.692×10^−4^	3.744×10^−4^	gamma

## Abbreviations

The following abbreviations are used in this manuscript:

SHM	structural health monitoring
TSMM	traditional static measurement methods
LVDT	linear variable differential transformers
CPS	connected pipe systems
GNSS	global navigation satellite system
PT	photogrammetric techniques
LDT	laser displacement technologies
ABC	Artificial Bee colony Algorithm
BN	Bayesian network
DBN	Dynamic Bayesian network
MS	mode shape
MNMS	mass normalized mode shape
GA	Genetic Algorithm
PSO	Particle Swarm Optimization
*Fre*	modal frequency
$X_{Tem}$	temperature observed data
$X_{Hum}$	humidity observed data
$\beta_{0},\beta_{Tem},\beta_{Hum}$	the regression parameters
DAG	directed acyclic graph
*Tem*	environmental temperature
*Hum*	environmental humidity
*Def*	bridge deflection
MCMC	Markov Chain Monte Carlo
T&H	temperature and humidity sensor
COV	coefficient of variant
RM0	zero order polynomial
RM1	first order polynomial
RM2	second order polynomial
FP	failure probability
RI	reliability index
MC	Monte Carlo
SP	sensitivity parameter
